# Visceral adipose tissue and cardiometabolic risk factors in young Hispanic and non-Hispanic girls

**DOI:** 10.3389/fped.2022.892206

**Published:** 2022-09-12

**Authors:** Victoria L. Bland, Joseph M. Kindler, Robert M. Blew, Kristin E. Morrill, Denise J. Roe, Scott B. Going

**Affiliations:** ^1^School of Nutritional Sciences and Wellness, University of Arizona, Tucson, AZ, United States; ^2^Department of Nutritional Sciences, University of Georgia, Athens, GA, United States; ^3^Department of Health Promotion Sciences, University of Arizona, Tucson, AZ, United States; ^4^The University of Arizona Cancer Center, Tucson, AZ, United States; ^5^Department of Epidemiology and Biostatistics, University of Arizona, Tucson, AZ, United States

**Keywords:** adipose tissue, obesity, Hispanic, females, metabolic health

## Abstract

**Background:**

Risk factors for cardiometabolic diseases (e.g., type 2 diabetes, cardiovascular disease) can begin developing in childhood. Elevated body mass index (BMI) is associated with greater likelihood of developing such diseases; however, this relationship varies by race and ethnicity. Notably, Hispanics tend to have high rates of obesity and are disproportionately affected by type 2 diabetes. We aimed to determine if visceral adiposes tissue (VAT) is associated with cardiometabolic risk factors (i.e., triglycerides, cholesterol, insulin resistance, C-reactive protein, and blood pressure), independent of BMI percentile, in a sample of primarily Hispanic adolescent girls.

**Methods and results:**

A total of 337 girls (73% Hispanic) took part in the cross-sectional study. Hispanic girls generally had greater BMI percentile, VAT, and cardiometabolic risk factors compared to non-Hispanic girls. Multiple linear regression was used to assess the relationships between Dual-energy X-ray Absorptiometry (DXA)-derived VAT and cardiometabolic outcomes, controlling for BMI percentile (<85th percentile or ≥85th percentile), age, ethnicity (Hispanic/non-Hispanic), and Tanner stage. Significant interactions between VAT and BMI percentile were identified for almost all cardiometabolic outcomes. Upon stratification, the association between VAT and cardiometabolic outcomes was strongest in girls ≥85th BMI percentile, as compared to girls <85th percentile. However, VAT was only significantly associated with higher triglycerides (girls ≥85th percentile) and higher insulin resistance (both BMI percentiles) after stratification.

**Conclusion:**

VAT was associated with increased triglycerides and insulin resistance in girls with overweight or obesity. These findings warrant further investigation between VAT and cardiometabolic health in Hispanic adolescents who tend to accumulate more adipose tissue during adolescence.

## Summary

Cardiometabolic diseases, such as type 2 diabetes and cardiovascular disease, typically manifest during adulthood but have been increasingly diagnosed in adolescence. Visceral adipose tissue (VAT) has been linked with cardiometabolic diseases in adults; however, there is a paucity of literature assessing the relationship between VAT and cardiometabolic health in adolescents. Our study focused on girls identifying as Hispanic ethnicity, a population with elevated rates of obesity and type 2 diabetes. We aimed to assess the association between Dual-energy X-ray Absorptiometry-derived VAT and cardiometabolic outcomes (triglycerides, cholesterol, insulin resistance, C-reactive protein, and blood pressure) in 9–12 year-old girls (73% Hispanic), controlling for body mass index (BMI) percentile. Overall, we found the relationship between VAT and cardiometabolic outcomes was dependent on body mass index (BMI) percentile. In the fully adjusted BMI stratified (BMI, 85th percentile of ≥85th percentile) models, VAT was significantly associated with triglycerides (BMI >85th percentile only) and insulin resistance (both BMI groups). These findings align with prior research that has shown a significant relationship between VAT with fasting insulin and insulin sensitivity. We expand on the existing literature with our findings that VAT may increase cardiometabolic risk, independent of BMI percentile.

## Introduction

Cardiometabolic diseases, such as type 2 diabetes and cardiovascular disease, are typically considered adult-onset diseases; however, type 2 diabetes is increasingly diagnosed during adolescence and childhood obesity may put kids at an elevated risk for cardiovascular disease later in life (1). This is particularly relevant given the rising rates of overweight and obesity and the corresponding rise in cardiometabolic risk factors (CMRs) (e.g., triglycerides, insulin resistance) observed in adolescents (1, 2). In the United States, based on race and ethnicity, the highest rates of obesity are observed in Hispanic adults (47.0%) and adolescents (25.8%) (2) but there is large inter-individual variation in cardiometabolic health between individuals classified as obese based solely on BMI (3, 4).

BMI is a convenient tool to monitor population health and conduct risk stratification but is a surrogate measure for adiposity and cannot provide insight into adipose tissue distribution (5). Direct body composition assessment can provide a more in-depth understanding of the relationship between obesity and cardiometabolic health (3, 5). Fat accumulated in the abdominal region has been found to be a better predictor of cardiometabolic health than either BMI or percent body fat among adults (6). Among children and adolescents, changes in adiposity levels over time may not be fully captured when standard BMI categories are looked at alone which underscores the need to incorporate other body composition measures, such as regional fat depots (6). Notably, increases in adiposity have not been found to be the same across racial and ethnic subpopulations with Mexican American adolescents having greater increases in adiposity relative to their non-Hispanic White (NHW) and non-Hispanic Black (NHB) counterparts (6).

Visceral adipose tissue (VAT) has been associated with metabolic dysregulation and cardiovascular disease in adults but the relationship between VAT and cardiometabolic health in adolescents has not been well-established. Most studies in adolescents, particularly in Hispanic adolescents, have focused on the relationship between VAT and insulin sensitivity but few studies in adolescents have assessed the association of VAT with other CMRs (1, 7). The Soft Tissue And bone development in young giRls (STAR) study was an observational study designed to assess the relationship between body composition, CMRs, and bone in adolescent girls (73% Hispanic). In the STAR study, we previously found that anthropometric estimates of body fatness performed just as well as more precise Dual-energy X-ray Absorptiometry (DXA) measures of adiposity (percent body fat, fat mass index, android fat mass) for predicting CMRs (8); however, the influence of VAT has not been assessed in this group. Therefore, the purpose of this study was to expand on the limited research assessing VAT in Hispanic girls and investigate the association of VAT with a range of CMRs.

## Methods

### Study participants

This was a secondary analysis of cross-sectional data from the STAR study. Participants were 9–12-year-old girls recruited from local schools, pediatric clinics, and wellness community events in Tucson, AZ between 2013 and 2018 (ClinicalTrials.gov number, NCT02654262). Girls were excluded if they had a diagnosis of diabetes, were taking any medications that may affect body composition, or had a physical and/or learning disability which made the participant unable to comply with study protocols. The study was approved by the University of Arizona Human Subjects Protection Committee. Written informed assent was obtained from all participants and consent was obtained from their parents or legal guardians. Participants' guardians completed questionnaires to report age, health history (e.g., medical diagnoses, medications), race (American Indian/Alaska Native, Asian, Black or African American, Native Hawaiian or Other Pacific Islander, White, more than one race, or prefer not to answer) and Hispanic ethnicity (yes or no) of the participant. Pubertal status was determined by self-reported questionnaire where the girls indicated their breast and pubic hair development based on pictures depicting the Tanner stages of pubertal maturation (9).

### Anthropometric measures

Anthropometric measurements have been previously described (8). Body mass was measured to the nearest 0.1 kg using a calibrated scale (Seca, Model 881, Hamburg, Germany). Standing and sitting height were measured at full inhalation to the nearest mm using a stadiometer (Shorr Height Measuring Board, Olney, MD). BMI-for-age percentiles were used to classify girls as underweight (<5th percentile), normal weight (≥5 and <85th percentiles), overweight (≥85 and <95th percentiles), or obese (≥95th percentile) (10).

### Cardiometabolic risk factors

Full details regarding acquisition and analysis of CMRs have been previously described (8). In brief, serum was extracted from fasting blood samples and sent to a CLIA certified laboratory where measures of triglycerides, high-density lipoprotein cholesterol (HDL-C), low-density lipoprotein cholesterol (LDL-C), and glucose were obtained. Fasting insulin was measured at a University of Arizona laboratory and insulin resistance was estimated using the homeostatic model assessment of insulin resistance (HOMA-IR), calculated as: HOMA-IR = (insulin [μU/L] × glucose [mM/L])/22.5 (11). C-Reactive Protein (CRP) was measured in the serum using a Beckman Coulter AU5812 Clinical Chemistry Analyzer (12). An automated blood pressure monitor (Omron HEM-907XL) was used to measure systolic and diastolic blood pressure using the appropriately sized blood pressure cuff with subjects in the seated position after 15 min of rest. The average of three measurements was used.

### Dual-energy X-ray absorptiometry

As previously described (13), full body DXA (Prodigy and iDXA models; GE/Lunar Radiation Corp, Madison, WI) scans were performed by a single certified technician using standard subject positioning and data acquisition protocols. All scan analyses were completed using GE Lunar enCORE™ software version 16.2. From the whole-body DXA scans, an android region of interest (ROI) was defined as the region with its lower border placed at the top of the iliac crest and upper border defined by 20% of the distance between the top of the iliac crest and immediately below the chin. The ROI extended laterally to include the entire torso. DXA-derived VAT was obtained from the ROI using the GE Lunar CoreScan application. This application uses a validated algorithm to estimate visceral fat mass from the android region (14). DXA-derived VAT has been shown to correlate well with magnetic resonance imaging (MRI)-derived VAT from the L4-L5 region in our sample (*r* = 0.79, *p* < 0.001) (13) and with computed tomography (CT)-derived estimates of VAT in adolescents classified as overweight or obese (*r* = 0.86; 95% CI: 0.08, 0.90) (15).

### Statistical analysis

Descriptive statistics for participant characteristics were calculated for girls with complete data. Girls were stratified by ethnicity status (Hispanic or non-Hispanic) and between group differences were tested. Multiple linear regression analyses were used to assess the relationship between VAT and each CMR. Covariates included in all models were selected *a priori* based on the literature and included BMI percentile, age, ethnicity, and Tanner stage. To determine if the relationship between VAT and CMRs was dependent on BMI, we tested for an interaction between VAT and BMI percentile. Since the interaction of two continuous variables can be difficult to interpret, we stratified BMI percentile (<85 or ≥85th percentile) based statistically on model visuals and clinically as the cutoff for classification as “overweight.” Models with statistically significant interactions were then stratified by BMI percentile category and re-run, continuing to control for BMI percentile (continuous), age, ethnicity, and Tanner stage.

Due to the DXA scanner being upgraded to a new model partway through the study, 63 girls (~19%) were scanned on the iDXA while the remaining 274 girls were scanned on the Prodigy model. We conducted a sensitivity analysis by re-running all models using only girls scanned on the Prodigy DXA scanner. Non-normally distributed outcome variables (VAT, triglycerides, LDL-C, HDL-C, HOMA-IR, and CRP) were log transformed to achieve an approximately normal distribution. All models met the assumptions of linear regression (i.e., linearity, homoscedasticity, and normality) and did not have concerns with collinearity. All analyses were performed in R version 3.6.3 (R Foundation for Statistical Computing, Vienna, Austria). To correct for multiple tests a Bonferroni adjusted alpha level of 0.007 (α = 0.05/7 tests) was used to determine statistical significance.

## Results

Of the 356 girls that took part in the STAR cross-sectional study, 337 girls had complete data (missing data: *n* = 11 ethnicity, *n* = 6 blood biomarkers, *n* = 2 blood pressure). Sample characteristics are described in Table 1. Approximately 73% of the sample identified as Hispanic ethnicity. Girls identifying as Hispanic were significantly younger, had a higher BMI and BMI percentile, and had greater total fat mass and VAT compared to non-Hispanic girls. Hispanic girls also had higher triglycerides, HOMA-IR, and CRP, but there were no significant differences between Hispanic and non-Hispanic girls for HDL-C, LDL-C, or blood pressure.

**Table 1:** Sample characteristics of girls with complete data in the STAR study (*n* = 337).

	**All**	**Non-hispanic**	**Hispanic**	***P***
	**(*n* = 337)**	**(*n* = 92)**	**(*n* = 245)**	
Age (years)	10.76 (1.10)	10.96 (1.11)	10.69 (1.09)	0.043
Postmenarcheal [*n* (%)]	65 (19.3)	16 (17.4)	49 (20.0)	0.7
Race [*n* (%)]				<0.001
American Indian/Alaska Native	12 (3.6)	4 (4.3)	8 (3.3)	
Asian	5 (1.5)	4 (4.3)	1 (0.4)	
Black or African American	10 (3.0)	4 (4.3)	6 (2.4)	
More than one race	28 (8.3)	14 (15.2)	14 (5.7)	
Not Indicated	47 (14.0)	2 (2.2)	45 (18.4)	
White	235 (69.7)	64 (69.6)	171 (69.8)	
Tanner Stage [*n* (%)]				0.201
1	187 (55.5)	46 (50.0)	141 (57.5)	
2	108 (32.0)	30 (32.6)	78 (31.8)	
3	27 (8.0)	12 (13.0)	15 (6.1)	
4	11 (3.3)	4 (4.3)	7 (2.9)	
5	4 (1.2)	0 (0.0)	4 (1.6)	
Height (cm)	145.68 (9.64)	146.92 (10.12)	145.21 (9.43)	0.147
Weight (kg) (median [IQR])	41.30 [33.15, 52.30]	40.67 [32.77, 48.43]	41.75 [33.30, 52.75]	0.33
BMI (kg/m^∧^2) (median [IQR])	19.20 [16.70, 23.10]	18.30 [16.25, 21.63]	19.50 [17.00, 23.60]	0.019
BMI Percentile (median [IQR])	75.50 [41.00, 94.30]	64.40 [29.43, 91.53]	80.80 [48.00, 94.60]	0.007
BMI Category [*n* (%)]				0.033
Underweight (<5th percentile)	9 (2.7)	5 (5.4)	4 (1.6)	
Normal Weight (5th−85th percentile)	198 (58.8)	60 (65.2)	138 (56.3)	
Overweight (85th−95th percentile)	53 (15.7)	8 (8.7)	45 (18.4)	
Obese (≥95th percentile)	77 (22.8)	19 (20.7)	58 (23.7)	
Total body fat mass (kg) (median [IQR])	13008.52 [8823.72, 20285.59]	11287.05 [7859.83, 16652.48]	13690.44 [9496.40, 21065.23]	0.009
Body Fat Percentage	37.06 (8.70)	33.89 (8.76)	38.25 (8.38)	<0.001
Visceral fat (g) (median [IQR])	119.90 [53.20, 290.48]	92.61 [42.26, 167.05]	139.24 [55.74, 309.04]	0.01
Triglycerides (mg/dL) (median [IQR])	88.00 [65.00, 119.00]	82.00 [57.00, 107.00]	90.00 [68.00, 126.00]	0.049
HDL-C (mg/dL) (median [IQR])	51.00 [45.00, 59.00]	51.00 [45.00, 60.00]	51.00 [45.00, 58.00]	0.547
LDL-C (mg/dL) (median [IQR])	96.00 [80.00, 117.00]	94.50 [79.75, 120.00]	96.00 [81.00, 117.00]	0.945
HOMA-IR (median [IQR])	3.90 [2.80, 5.40]	3.45 [2.70, 4.90]	4.00 [3.00, 5.60]	0.041
CRP (mg/dL) (median [IQR])	0.40 [0.20, 1.40]	0.30 [0.20, 0.80]	0.40 [0.20, 1.50]	0.013
Systolic Blood Pressure (mmHg)	99.84 (8.88)	99.42 (9.77)	99.99 (8.54)	0.602
Diastolic Blood Pressure (mmHg)	63.52 (7.15)	62.80 (7.38)	63.79 (7.06)	0.261

Differences between ethnicity groups were tested using a two-sample t-test for normally distributed continuous variables, Wilcoxon rank-sum test for non-normally distributed continuous variables, and chi-squared test for categorical variables.

BMI, body mass index; CRP, c-reactive protein; HDL-C, high density lipoprotein cholesterol; HOMA-IR, homeostatic model assessment of insulin resistance; LDL-C, low density lipoprotein cholesterol.

Results from multiple linear regression models assessing the relationship between VAT and CMRs are presented in Table 2. Results from regression analyses involving VAT and CMRs, and the moderating effect of BMI percentile as a continuous variable, are presented in Supplementary Table 1. Regardless of whether BMI was considered as a continuous variable or categorical variable, BMI modified the association between VAT and CMRs similarly. A significant interaction between VAT and BMI percentile was identified for triglycerides, HOMA-IR, CRP, systolic blood pressure, and diastolic blood pressure. There was no significant (*p* < 0.007) interaction observed for HDL-C (*p* = 0.015) or LDL-C (*p* = 0.08).

**Table 2:** Multiple linear regression models assessing the relationship between visceral adipose tissue and cardiometabolic risk factors.

		**Interaction models**			**Stratified models**			
		**All Girls (*****n*** = **337)**			**BMI** < **85th Per (*****n*** = **207)**		**BMI** ≥ **85th Per (*****n*** = **130)**	
		**Coefficient (SE)**	***P***		**Coefficient (SE)**	***P***	**Coefficient (SE)**	***P***
Triglycerides (log)	Age	0.048 (0.025)	0.05	Age	0.070 (0.032)	0.03	0.023 (0.042)	0.59
	Tanner stage	−0.031 (0.032)	0.33	Tanner Stage	−0.031 (0.046)	0.50	−0.052 (0.047)	0.27
	Hisp. ethnicity	0.056 (0.053)	0.29	Hisp. Ethnicity	0.046 (0.064)	0.47	0.061 (0.096)	0.53
	BMI: ≥85th per.	−1.211 (0.381)	0.002	BMI percentile	0.002 (0.001)	0.16	0.005 (0.014)	0.73
	VAT (log)	0.043 (0.029)	0.14	VAT (log)	0.021 (0.032)	0.50	**0.279 (0.085)**	**0.001**
	VAT*BMI inter	**0.247 (0.068)**	**<0.0001**					
Low density lipoprotein cholesterol (LDL-C) (log)	Age	−0.012 (0.015)	0.41	Age	−0.009 (0.020)	0.67	−0.008 (0.025)	0.76
	Tanner stage	−0.001 (0.020)	0.97	Tanner Stage	−0.012 (0.029)	0.68	0.001 (0.028)	0.97
	Hisp. Ethnicity	−0.029 (0.033)	0.37	Hisp. Ethnicity	−0.032 (0.040)	0.43	−0.024 (0.057)	0.68
	BMI: ≥85th per.	−0.339 (0.235)	0.15	BMI percentile	0.001 (0.001)	0.34	0.004 (0.008)	0.62
	VAT (log)	0.016 (0.018)	0.38	VAT (log)	0.008 (0.020)	0.68	0.069 (0.051)	0.18
	VAT*BMI Inter	0.071 (0.042)	0.09					
High density lipoprotein cholesterol (HDL-C) (log)	Age	−0.011 (0.011)	0.34	Age	−0.026 (0.015)	0.09	0.003 (0.017)	0.85
	Tanner stage	0.005 (0.014)	0.75	Tanner Stage	0.003 (0.022)	0.90	0.025 (0.019)	0.19
	Hisp. ethnicity	0.010 (0.024)	0.67	Hisp. Ethnicity	−0.009 (0.030)	0.77	0.043 (0.038)	0.27
	BMI: ≥85th per.	0.333 (0.171)	0.05	BMI percentile	−0.001 (0.001)	0.22	−0.014 (0.005)	0.01
	VAT (log)	−0.028 (0.013)	0.03	VAT (log)	−0.018 (0.015)	0.23	−0.055 (0.034)	0.10
	VAT*BMI Inter	−0.074 (0.030)	0.015					
HOMA-IR (log)	Age	0.048 (0.023)	0.04	Age	**0.079 (0.028)**	**0.005**	0.022 (0.042)	0.60
	Tanner stage	0.038 (0.030)	0.20	Tanner Stage	0.017 (0.040)	0.66	0.018 (0.047)	0.70
	Hisp. ethnicity	0.030 (0.049)	0.53	Hisp. Ethnicity	0.061 (0.055)	0.27	−0.059 (0.095)	0.54
	BMI: ≥85th per.	−1.535 (0.351)	<0.0001	BMI percentile	**0.004 (0.001)**	**0.001**	0.008 (0.014)	0.57
	VAT (log)	0.098 (0.027)	<0.0001	VAT (log)	0.057 (0.027)	0.04	**0.408 (0.084)**	**<0.0001**
	VAT*BMI inter	**0.327 (0.062)**	**<0.0001**					
C-reactive protein (CRP) (log)	Age	−0.064 (0.064)	0.32	Age	0.000 (0.085)	0.99	−0.086 (0.104)	0.41
	Tanner stage	−0.131 (0.084)	0.12	Tanner Stage	−0.081 (0.1210	0.50	−0.313 (0.117)	0.008
	Hisp. ethnicity	0.109 (0.138)	0.43	Hisp. Ethnicity	0.093 (0.167)	0.58	0.156 (0.237)	0.51
	BMI: ≥85th per.	−2.439 (0.995)	0.02	BMI percentile	0.008 (0.004)	0.03	0.082 (0.034)	0.017
	VAT (log)	0.117 (0.075)	0.12	VAT (log)	0.021 (0.084)	0.81	0.406 (0.210)	0.06
	VAT*BMI Inter	**0.595 (0.177)**	**0.001**					
Systolic blood pressure	Age	**1.618 (0.484)**	**0.001**	Age	1.587 (0.659)	0.02	**2.479 (0.737)**	**0.001**
	Tanner stage	0.612 (0.629)	0.33	Tanner Stage	0.276 (0.944)	0.77	0.329 (0.831)	0.69
	Hisp. ethnicity	0.459 (1.034)	0.66	Hisp. Ethnicity	1.162 (1.306)	0.37	0.137 (1.6870)	0.94
	BMI: ≥85th per.	−22.644 (7.468)	0.003	BMI percentile	−0.001 (0.028)	0.97	**0.847 (0.240)**	**0.001**
	VAT (log)	−0.274 (0.565)	0.63	VAT (log)	−0.256 (0.652)	0.70	0.791 (1.495)	0.60
	VAT*BMI Inter	**4.580 (1.326)**	**0.001**					
Diastolic blood pressure	Age	**1.168 (0.394)**	**0.003**	Age	1.075 (0.518)	0.04	1.614 (0.661)	0.02
	Tanner stage	−0.180 (0.512)	0.73	Tanner Stage	0.065 (0.742)	0.93	−0.802 (0.744)	0.28
	Hisp. ethnicity	0.673 (0.843)	0.43	Hisp. Ethnicity	1.313 (1.027)	0.20	−0.219 (1.511)	0.89
	BMI: ≥85th per.	−14.710 (6.084)	0.02	BMI percentile	0.005 (0.022)	0.80	0.406 (0.215)	0.06
	VAT (log)	0.035 (0.461)	0.94	VAT (log)	−0.084 (0.513)	0.87	1.529 (1.340)	0.26
	VAT*BMI Inter	**3.107 (1.080)**	**0.004**					

Unstandardized regression coefficients. BMI percentile as a binary variable in interaction models (BMI < 85th percentile as reference category) and as a continuous variable in stratified models. Reference category for ethnicity is non-Hispanic.

Bold text indicates statistical significance (p < 0.007).

BMI body mass index, HOMA-IR homeostatic model of insulin resistance, VAT visceral adipose tissue.

**Figure 1:**
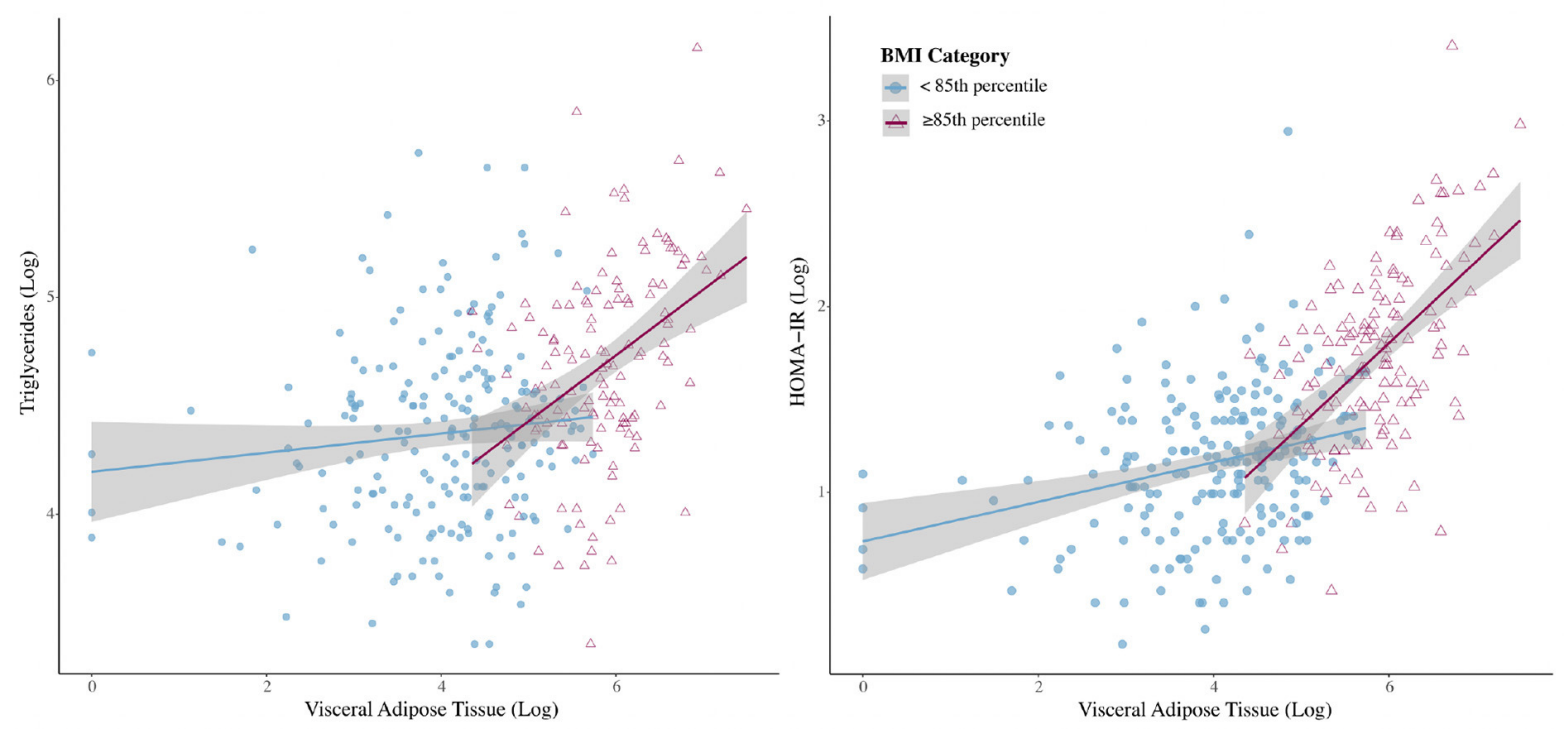
Interactions with 95% confidence intervals between BMI category and visceral adipose tissue for outcomes of triglycerides and homeostatic model of insulin resistance (HOMA-IR).

Linear regression models were then stratified by BMI percentile (<85 or ≥85th percentile) (Table 2). The relationship between VAT and CMRs was stronger in girls ≥85th percentile for BMI than it was for girls <85th percentile. However, after controlling for age, ethnicity, Tanner stage, and BMI percentile (continuous), VAT was only significantly associated with higher triglycerides in girls with a BMI ≥85th percentile and higher HOMA-IR in both BMI percentile groups (Figure 1). VAT explained ~6.7% of the variance in triglycerides and 12.5% of the variance in HOMA-IR for girls classified as overweight or obese. Results from the sensitivity analysis (Supplementary Table 2) did not change study conclusions.

## Discussion

While the relationship between VAT and cardiometabolic health has been well-established in adults, it is less understood in adolescents. To our knowledge, this is the first study to assess the influence of VAT on a variety of cardiometabolic risk factors in a sample of primarily Hispanic (70% Hispanic) adolescent girls. In our sample of adolescent girls, we found that the relationship between VAT and CMRs was dependent on BMI percentile. Upon stratification by BMI percentile (<85 or ≥85th percentile), the relationship between VAT and CMRs was strongest in girls above the 85th BMI percentile; however, this relationship was only statistically significant for the outcomes of triglycerides and HOMA-IR, after controlling for age, ethnicity, Tanner stage, and BMI percentile. These findings suggest that VAT confers a risk to certain CMRs beyond what BMI estimates in girls classified as overweight and obese. The relationship between VAT and CMRs did not differ by ethnicity. These findings build on an earlier analysis conducted in a portion of this group of girls (*n* = 288) which concluded that indirect measures of adiposity (BMI percentile, BMI, waist circumference, and waist-to-hip ratio) performed as well as DXA-derived measures of adiposity (percent body fat, android fat mass, and fat mass index) to predict cardiometabolic risk factors (8). However, with the inclusion of VAT, we found that VAT explains variance in HOMA-IR and triglycerides, even after controlling for BMI.

In our sample, the higher rates of obesity among Hispanic vs. non-Hispanic girls were in line with findings from population-based studies (2) and corresponded to higher total body fat mass, percent body fat, triglycerides, HOMA-IR, and CRP. Prior research has shown that ethnicity influences trunk fat accumulation, HOMA-IR, triglyceride levels, and liver steatosis prevalence among children and adolescents (16), but after controlling for the observed differences in BMI percentile and visceral fat, we found ethnicity to be non-significant in our models. This suggests that differences in CMRs observed between Hispanic and non-Hispanic girls is likely due to differences in adiposity and is not directly related to ethnicity, *per se*.

Prior studies in Hispanic children and adolescents have focused on the relationship between VAT and insulin regulation (1). In a study of 32 Hispanic children (38% female, BMI ≥85th percentile), regression analyses indicated that VAT was associated with greater fasting insulin, lower insulin sensitivity, and greater insulin resistance, independent of total fat mass (17). While we only assessed a metric of insulin resistance in our study, we also found VAT to be associated with increased insulin resistance in Hispanic girls with a BMI ≥85th percentile. Similar to Cruz et al. (17), Gower et al. (7) also assessed the relationship between VAT with fasting insulin and insulin sensitivity among prepubertal children (43% female, NHW and NHB sample) but expanded outcomes to include triglyceride concentration and HDL-C. Similar to our findings, Gower and colleagues showed that VAT was independently associated with triglycerides after controlling for total fat, sex, and race (7). Additionally, they found VAT was associated with fasting insulin (7). Notably, Gower et al. (7) observed a significant association with fasting insulin, but not insulin sensitivity, while we observed a significant association with HOMA-IR in girls classified as overweight and obese. Further exploration of the relationship between VAT and insulin regulation and the potential effect of ethnicity among adolescents is warranted.

We are not the first to identify an association of VAT with insulin resistance and triglycerides in adolescents, and potential underlying mechanisms of action have been proposed to explain this relationship. Briefly, one hypothesis is the “portal theory,” where the breakdown of visceral fat through a series of steps leads to an increase of gluconeogenesis in the liver and low output of very low density lipoprotein resulting in impaired glucose tolerance and elevated triglycerides (1, 18). An alternative hypothesis is that products from visceral fat (e.g., adipokines) and cortisol production within the VAT could influence metabolic health (1, 18). Finally, it is also possible that VAT is associated with ectopic fact deposition in other organs (e.g., liver, heart, pancreas) that could also be associated with metabolic dysfunction (1). However, while our manuscript and prior cited literature has focused on the influence of VAT on cardiometabolic health, the importance of lean mass cannot be discounted. A recent systematic review and meta-analysis by Córdoba-Rodríguez and colleagues showed that children and adolescents with insulin resistance and metabolic syndrome had a lower proportion of lean and fat free mass (19). Therefore, the potentially negative influence of VAT and favorable influence of lean mass on metabolic health warrants further exploration.

One limitation of this study was the cross-sectional design. Given the significant changes in adiposity through adolescence, longitudinal studies are needed to better understand how adiposity over time influences cardiometabolic outcomes. Second, DXA-derived VAT has not been well-validated in children and adolescents; however, it has been shown to be highly correlated with the gold standards of CT and MRI measures of VAT in adolescents, including within our own sample (13, 15). Third, there can be significant heterogeneity between individuals of Hispanic ethnicity depending on country of origin and we did not collect this data in the STAR study. Fourth, we cannot discount the fact that the disproportionate number of Hispanic girls to non-Hispanic girls in our study sample may have limited our ability to detect an interaction between VAT and ethnicity. Finally, transient insulin resistance can occur during mid-to-late puberty in adolescents (20) which could have influenced findings; however, the majority of our girls were in pre-to-early puberty and we accounted for this potential limitation by controlling for Tanner stage.

Hispanic adolescents have higher rates of overweight and obesity than other racial and ethnic subpopulations (2), tend to gain more body fat during development than NHW and NHB adolescents (6), and are more prone to abdominal fat deposition (16). Beyond the cardiovascular risks associated with excess adiposity, individuals of Hispanic ethnicity are at an elevated risk of type 2 diabetes (21) and non-alcoholic fatty liver disease (22). In this analysis, we observed an association between VAT and insulin resistance and triglycerides in young girls classified as overweight and obese. These findings suggest that CMRs may begin to develop in adolescence and VAT increases the risk in girls with overweight and obesity, independent of BMI percentile; however, further research to understand the relationship of VAT with glucose regulation and triglycerides in adolescent Hispanic populations is warranted. Additionally, since VAT varies significantly with age, sex, race/ethnicity during the growing years, studies are needed to determine the most appropriate methods to account for these factors in research and clinical settings (e.g., pediatric growth charts). Lastly, more research is needed to understand how the relationship between VAT and CMRs changes over time in pediatric populations. Findings from these studies would inform the identification of individuals at risk for non-communicable diseases and interventions to decrease disease risk early on in life.

## Data availability statement

The raw data supporting the conclusions of this article will be made available by the authors, without undue reservation.

## Ethics statement

The studies involving human participants were reviewed and approved by University of Arizona Human Subjects Protection Committee. Written informed consent to participate in this study was provided by the participants' legal guardian/next of kin.

## Author contributions

SG and DR contributed to conception and design of the study. RB, VB, and SG were involved in data acquisition. VB, JK, RB, and DR designed and performed the statistical analysis. VB and KM wrote the first draft of the manuscript. All authors contributed to manuscript revision, read, and approved the submitted version.

## Funding

This study was funded by National Institute of Child Health and Human Development (Award #HD074565).

## Conflict of interest

The authors declare that the research was conducted in the absence of any commercial or financial relationships that could be construed as a potential conflict of interest.

## Publisher's note

All claims expressed in this article are solely those of the authors and do not necessarily represent those of their affiliated organizations, or those of the publisher, the editors and the reviewers. Any product that may be evaluated in this article, or claim that may be made by its manufacturer, is not guaranteed or endorsed by the publisher.
